# Compromised anti-tumor–immune features of myeloid cell components in chronic myeloid leukemia patients

**DOI:** 10.1038/s41598-021-97371-8

**Published:** 2021-09-10

**Authors:** Ibuki Harada, Haruka Sasaki, Koichi Murakami, Akira Nishiyama, Jun Nakabayashi, Motohide Ichino, Takuya Miyazaki, Ken Kumagai, Kenji Matsumoto, Maki Hagihara, Wataru Kawase, Takayoshi Tachibana, Masatsugu Tanaka, Tomoyuki Saito, Heiwa Kanamori, Hiroyuki Fujita, Shin Fujisawa, Hideaki Nakajima, Tomohiko Tamura

**Affiliations:** 1 Department of Immunology, Yokohama City University Graduate School of Medicine, 3-9 Fukuura, Kanazawa-ku, Yokohama, 236-0004 Japan; 2 Advanced Medical Research Center, Yokohama City University, Kanagawa, Japan; 3 Department of Stem Cell and Immune Regulation, Yokohama City University Graduate School of Medicine, Kanagawa, Japan; 4 Department of Orthopaedic Surgery, Yokohama City University School of Medicine, Kanagawa, Japan; 5 Department of Hematology, Kanagawa Cancer Center, Kanagawa, Japan; 6 Department of Hematology, Saiseikai Yokohama Nanbu Hospital, Kanagawa, Japan; 7 Department of Hematology, Yokohama City University Medical Center, Kanagawa, Japan; 8 Present Address: College of Liberal Arts and Sciences, Mathematics, Tokyo Medical and Dental University, Tokyo, Japan; 9 Present Address: Yokohama Brain and Spine Center, Kanagawa, Japan

**Keywords:** Cancer, Computational biology and bioinformatics, Immunology, Diseases, Medical research, Oncology, Pathogenesis

## Abstract

Chronic myeloid leukemia (CML) is a form of myeloproliferative neoplasm caused by the oncogenic tyrosine kinase BCR-ABL. Although tyrosine kinase inhibitors have dramatically improved the prognosis of patients with CML, several problems such as resistance and recurrence still exist. Immunological control may contribute to solving these problems, and it is important to understand why CML patients fail to spontaneously develop anti-tumor immunity. Here, we show that differentiation of conventional dendritic cells (cDCs), which are vital for anti-tumor immunity, is restricted from an early stage of hematopoiesis in CML. In addition, we found that monocytes and basophils, which are increased in CML patients, express high levels of PD-L1, an immune checkpoint molecule that inhibits T cell responses. Moreover, RNA-sequencing analysis revealed that basophils express genes related to poor prognosis in CML. Our data suggest that BCR-ABL not only disrupts the “accelerator” (i.e., cDCs) but also applies the “brake” (i.e., monocytes and basophils) of anti-tumor immunity, compromising the defense against CML cells.

## Introduction

Chronic myeloid leukemia (CML) is a myeloproliferative neoplasm associated with the *BCR-ABL1* fusion oncogenes generated upon t(9;22) chromosomal translocations in hematopoietic stem cells (HSCs)^1^. *BCR-ABL1* encodes a constitutively active BCR-ABL tyrosine kinase. This dysregulated tyrosine kinase transforms the cells and leads to enhanced proliferation and genomic instability of CML cells, along with suppressed apoptosis. CML is known to progress through three phases: chronic phase (CML-CP), accelerated phase (CML-AP), and blast crisis (CML-BC). CML-CP typically lasts 3 to 5 years, and as additional genetic lesions accumulate, the disease eventually progresses to CML-AP, and CML-BC with an extremely poor prognosis^2^. The development of tyrosine kinase inhibitors (TKIs) has dramatically improved the prognosis of CML. Currently, the life expectancy of a newly diagnosed patient with CML-CP is close to normal. However, several issues remain, such as intolerance or a decreased quality of life due to early and late toxicity of TKIs, development of resistant mutations to TKIs, and increased financial burden^3,4^. Therefore, to find clues to solve the problems, deeper understanding of CML pathogenesis is needed.

The immune system plays critical roles in tumor pathogenesis^5^. Recently, rapid clinical progress of cancer immunotherapy with chimeric antigen receptor-modified T cells, or immune modulation using antibodies targeting the programmed death 1 (PD-1)–programmed death-ligand 1 (PD-L1) pathway, has underscored the importance of anti-tumor immunity in cancer therapy^6–8^. As for CML, the immune system has long been implicated in controlling CML, as evidenced by the effects of graft-versus-leukemia response–based therapies such as allogeneic hematopoietic stem cell transplantation, donor lymphocyte infusion, and interferon alpha (IFNα)^9–12^. Thus, immunological control may be an attractive option to completely cure CML. However, CML patients do not spontaneously develop effective anti-tumor immunity^13,14^. The bone marrow (BM) of patients with CML have an immunosuppressive tumor microenvironment^15^. Both CD4^+^ and CD8^+^ T cells in CML BM express high levels of putative exhaustion markers such as PD-1, T cell immunoglobulin mucin-3 (TIM3), and cytotoxic T lymphocyte associated antigen 4 (CTLA-4)^15^. The expansion of myeloid-derived suppressor cells (MDSCs) and regulatory T cells (Tregs) in both the BM and peripheral blood (PB), as well as, cytokine-mediated downregulation of MHC-II in CML progenitor cells, also facilitate evasion of host immune surveillance^13,16^. In addition, CML patients have defects in generating dendritic cells (DCs)^17,18^. Among conventional DCs, classically called myeloid DCs, type 1 cDCs (cDC1s) are potent antigen-presenting cells that play a pivotal role in inducing cytotoxic T lymphocyte (CTL) responses^19–21^. In CML patients, a marked reduction in the number of cDC1s is observed at diagnosis^18^. Although we previously reported that BCR-ABL strongly inhibits cDC development from an early stage of haematopoiesis in a mouse CML model^22^, the effects on differentiation and function of myeloid cells, including cDC1s, in CML patients remains unclear.

In this study, we analyzed BM progenitor cells in newly diagnosed CML patients and revealed that cDC differentiation is perturbed from an early progenitor stage of myelopoiesis due to downregulated interferon regulatory factor-8 (IRF8), a transcription factor essential for the development of cDC1s^19,23^. In addition, RNA-sequencing (RNA-seq) analysis of multiple myeloid cell fractions indicated that CML neutrophils harbor immunosuppressive features, such as enhanced expression of reactive oxygen species (ROS)-related genes. Moreover, monocytes and basophils, which are significantly increased in CML patients, were found to express high levels of PD-L1, suggesting that these cells may suppress anti-tumor immunity. Taken together, our data suggest that BCR-ABL may impair anti-tumor immunity against CML cells by disrupting cDC development and promoting myeloid cell-mediated immune suppression.

## Results

### cDC differentiation is perturbed from an early stage of haematopoiesis in CML

To investigate how myeloid cell differentiation is changed in CML, we first analyzed PB samples from 18 newly diagnosed CML patients. Samples from 6 healthy volunteers served as controls. The characteristics of the patients and healthy volunteers recruited in this study are shown in Table 1.

**Table 1:** Patient characteristics: (a) Patients and control individuals for peripheral white blood cell (WBC) analysis, (b) Patients and control individuals for bone marrow cells analysis.

	Sample, n	Age, median (range), years	Sex, Male, n	Sex, Female, n	WBC, median (range), cell counts/uL
**(**a**)**					
CML patients	18	56 (23–86)	12	6	48,550 (16,500–181,000)
Healthy Ctrl	6	48 (34–57)	6	0	5,200 (3,290–8,740)
**(b)**					
CML patients	12	56 (23–86)	8	4	
Normal Ctrl	4	78 (60–84)	2	2	

*n* numbers, *y* years-old.

**Figure 1:**
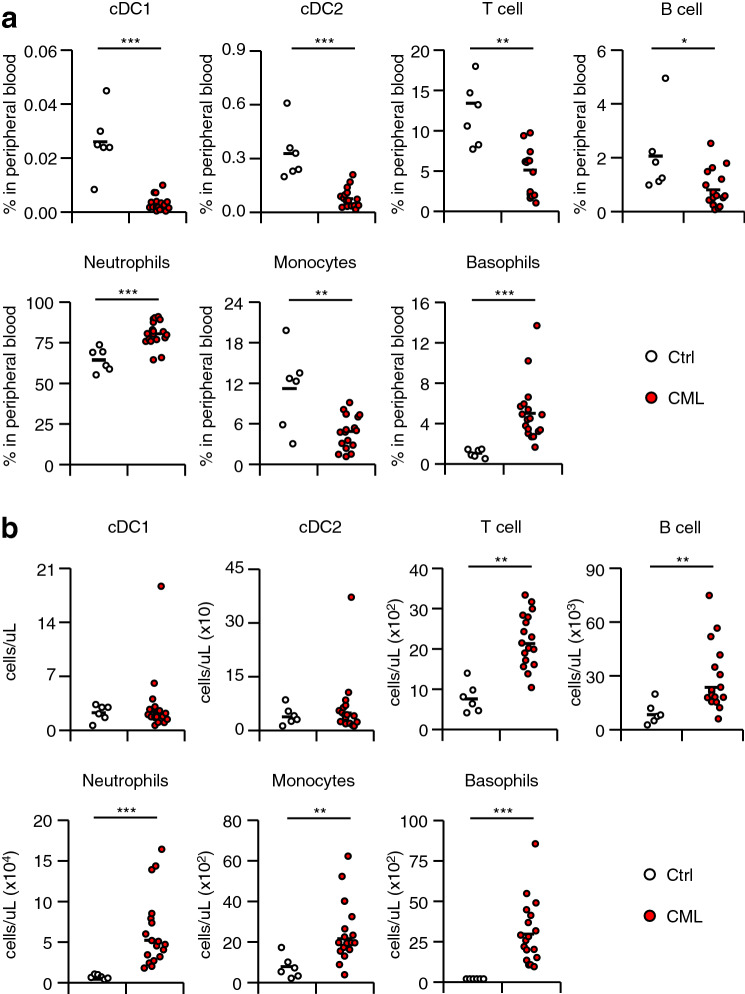
Proportion and absolute number of immune cells in CML patients. (**a**) Proportion of peripheral blood immune cells in CML patients (Healthy controls [Ctrl], n = 6; CML patients [CML], n = 18). (**b**) Absolute number of peripheral blood immune cells in CML patients (Healthy controls [Ctrl], n = 6; CML patients [CML], n = 18). The horizontal lines indicate mean values. *P < 0.05, **P < 0.01, ***P < 0.001 (Student’s *t*-test).

Flow cytometry analysis showed that both the percentages and absolute numbers of neutrophils and basophils were significantly increased in patients with CML (Fig. 1 and Supplementary Fig. S1a). In contrast, the percentages of monocytes, B cells, and T cells were decreased from half to a third of those in healthy volunteers, although their absolute numbers were increased. Notably, a severe reduction in the percentages of both cDC1s and cDC2s was observed in CML patients. These data were consistent with previous reports^17,18^.

**Figure 2:**
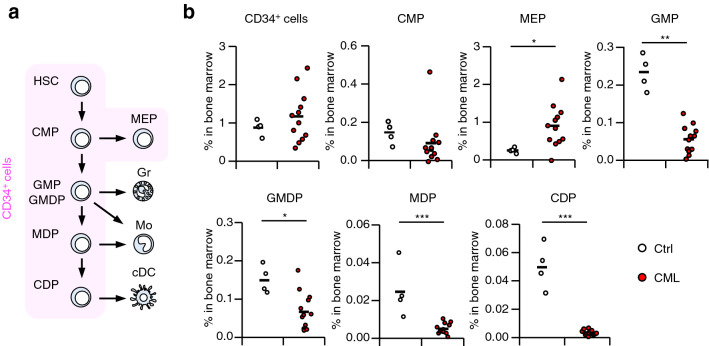
Proportion of myeloid progenitors in CML patients. (**a**) Schematic diagram of myeloid cell development. *HSC* hematopoietic stem cells, *CMP* common myeloid progenitors, *MEP* megakaryocyte erythroid progenitors, *GMP* granulocyte monocyte progenitors, *GMDP* granulocyte monocyte DC progenitors, *MDP* monocyte DC progenitors, *CDP* common dendritic cell progenitors; *Gr* granulocytes, *Mo* monocytes; *cDC* conventional dendritic cells including cDC1 and cDC2. (**b**) Proportion of bone marrow myeloid progenitors in CML patients (Normal controls [Ctrl], n = 4; CML patients [CML], n = 12). The horizontal lines indicate mean values. *P < 0.05, **P < 0.01, ***P < 0.001 (Student’s *t*-test).

We previously demonstrated that BCR-ABL potently inhibits differentiation of cDCs from an early stage of haematopoiesis by downregulating *IRF8* in a mouse CML model^22^. Therefore, we assumed that the dramatic decrease in the percentages of cDCs in human CML was also due to perturbed differentiation of myeloid cells. All myeloid cells, except for some tissue-resident macrophages and mast cells, are thought to be derived from BM HSCs (Fig. 2a)^24,25^. In humans, HSCs give rise to common myeloid progenitors (CMPs), which produce granulocyte-monocyte progenitors (GMPs), granulocyte-monocyte–DC progenitors (GMDPs), and megakaryocyte-erythroid progenitors (MEPs)^25,26^. GMPs and GMDPs differentiate into granulocytes, such as neutrophils, eosinophils, basophils, or mononuclear phagocytes, such as monocytes and DCs, although these pathways are still controversial. cDCs are generated from GMPs via monocyte-dendritic cell progenitors (MDPs) and common dendritic cell progenitors (CDPs), while monocytes are developed from both GMPs and MDPs^23,26,27^. Therefore, we analyzed the composition of hematopoietic progenitor populations in the BM of newly diagnosed CML patients (Fig. 2b and Supplementary Fig. S1b). For normal controls, BM aspiration samples from orthopedic patients with no hematological disorders were collected during surgical procedures (Table 1). The percentages of total CD34^+^ cells and CMPs in CML patients were comparable to those in normal controls. However, the percentages of GMPs and MDPs, and to a greater extent, CDPs, were severely reduced in patients with CML. In contrast, the percentage of MEPs was increased in CML patients. Overall, these results indicate that cDC-lineage development is disrupted from an early stage of myeloid cell differentiation in CML.

### IRF8 expression is downregulated in CML HSPCs

**Figure 3:**
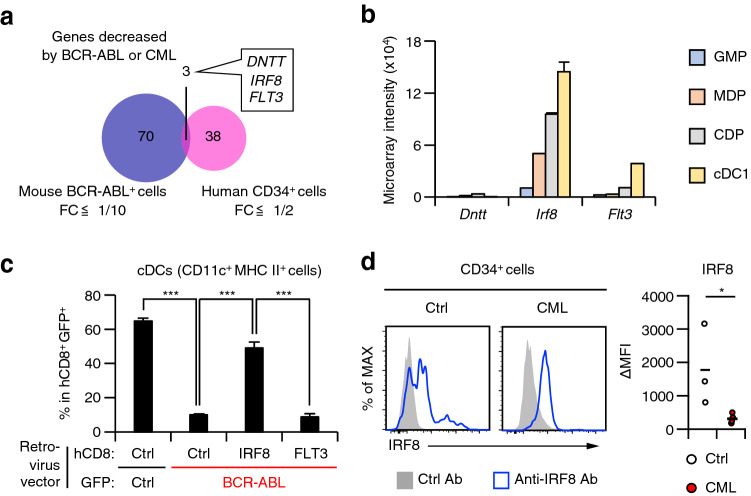
IRF8 expression in progenitors of CML patients. (**a**) A Venn diagram showing the overlap of genes downregulated in mouse bone marrow Lin^−^ cells transduced with BCR-ABL in vitro for 3 days and in CD34^+^ cells of CML patients. FC, fold change. (**b**) Normalized microarray intensities of *Dntt*, *Irf8*, and *Flt3* in mouse DC lineage progenitors. (**c**) Proportion of cDCs in in vitro mouse DC culture co-transduced with BCR-ABL and IRF8 or FLT3 (n = 6). (**d**) IRF8 protein expression in human CD34^+^ cells (Normal controls [Ctrl], n = 3; CML patients [CML], n = 4). Data were retrieved from GSE5550 (**a**), GSE44920 (**a**), and GSE84509 (**b**) and analysed. Values in (**b**) and (**d**) are the mean ± SD. The horizontal lines in (**d**) indicate mean values. *P < 0.05, **P < 0.01, ***P < 0.001 (Student’s *t*-test).

cDC development is impaired in CML in both mice and humans^17,18,22^. We speculated that a common mechanism may exist between these two species. Previously, we obtained transcriptomic data from mouse BM hematopoietic stem and progenitor cells (HSPC) transduced with a BCR-ABL retrovirus for 3 days in the presence of the DC-inducing cytokine FMS-like tyrosine kinase 3 ligand (FLT3L)^22^. We compared these data with publicly available data from BM CD34^+^ cells from CML patients^28^. By focusing on genes downregulated by BCR-ABL or in CML patients compared to mock-transduced cells or healthy controls, we found three common genes: *DNTT*, *IRF8*, and *FLT3* (Fig. 3a). We then analyzed expression of these genes during normal mouse DC development using our previously published microarray data (Fig. 3b). We found that expression of *Irf8* and *Flt3* gradually increased as the cells differentiated from GMPs to cDC1s. In contrast, *DNTT* was barely expressed during DC development. The same trend was observed in human DC progenitors, with the highest expression of *IRF8* in CDPs (Supplementary Fig. S2a,b).

Several reports have shown that both IRF8 and FLT3 are crucial for cDC development^19,20,29,30^. We previously demonstrated that BCR-ABL-mediated suppression of *IRF8* is the cause of cDC deficiency using mouse cells^22^. To compare the significance of IRF8 downregulation and that of FLT3 downregulation in cDC development in CML, we transduced a bicistronic retrovirus harboring *IRF8* or *FLT3* and human CD8t (hCD8t) into mouse BM lineage-negative cells, together with another bicistronic retrovirus encoding *BCR-ABL1* and green fluorescent protein (GFP). The cells were cultured in the presence of FLT3L (Fig. 3c). Consistent with our previous report, BCR-ABL impaired cDC development, which was restored by exogenous *IRF8* expression. In contrast, exogenous *FLT3* expression did not recover cDC development. Notably, while exogenous IRF8 partially restored *FLT3* expression, exogenous FLT3 did not affect *IRF8* expression (Supplementary Fig. S2c,d). These results suggest that IRF8 is particularly critical as a target of BCR-ABL to disrupt cDC differentiation in CML.

We also analyzed IRF8 protein expression levels in CML HSPCs. Intracellular staining of IRF8 revealed that the proportion of cells with high IRF8 expression levels and the mean IRF8 expression level were diminished in CML HSPCs compared to healthy controls (Fig. 3d). We have previously shown that IRF8 potently suppresses neutrophil production^31,32^, and in fact, *IRF8*^–/–^ mice develop a CML-like neutrophilia^33^. Thus, our results support the notion that BCR-ABL suppresses IRF8 at the hematopoietic progenitor stages not only to impede cDC development, but also to promote neutropenia in CML.

### Aberrant gene expression profile of neutrophils in CML patients

Recent reports suggest that tumor-induced myeloid cells, such as granulocytes and monocytes, interfere with anti-tumor immune responses by expressing various immunosuppressive molecules acting against cytotoxic CD8^+^ T cells^34–36^. Moreover, some reports also show that tumor-infiltrating DCs have defects in their functions, such as antigen presentation^37^.

**Figure 4:**
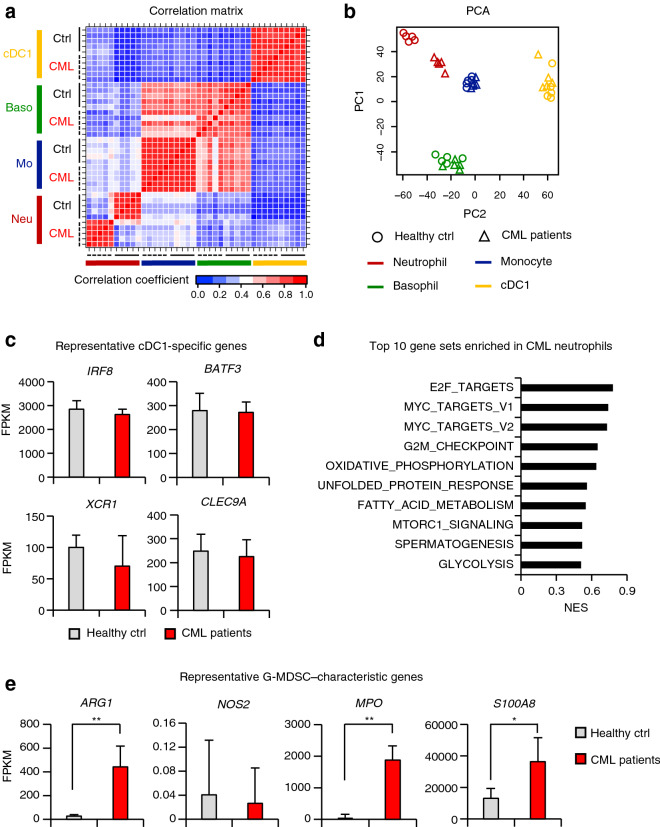
Transcriptomic analysis of myeloid cells in CML patients. (**a**) Pearson’s correlation coefficients of RNA-seq data between indicated cell populations (Healthy ctrl [Ctrl], n = 5; CML patients [CML], n = 5). Neu, neutrophils; Mo, monocytes; Baso, basophils; cDC1, type 1 conventional dendritic cells. (**b**) Principal component analysis (PCA) of RNA-seq data. (**c**) Expression of representative cDC1-specific genes in cDC1s from healthy ctrl and CML patients. (**d**) Normalized enrichment scores (NES) of top 10 gene sets enriched in neutrophils from CML patients compared to those from healthy ctrl identified using gene set enrichment analysis (GSEA). (**e**) Expression of representative G-MDSC–characteristic genes in neutrophils from healthy ctrl and CML patients. Values in (**c**) and (**e**) are the mean ± SD. *P < 0.05, **P < 0.01 (Student’s *t*-test).

To test whether myeloid cells in CML have any altered characteristics, we performed RNA-seq analyses of cDC1s, basophils, monocytes, and neutrophils isolated from PB of CML patients and healthy donors. Pearson’s correlation analysis and principal component analysis (PCA) showed that the gene expression profile of remaining cDC1s in CML patients displayed no significant differences compared to cDC1s in healthy donors (Fig. 4a,b). One possible reason may be that *BCR-ABL1* expression is very low in cDC1s (Supplementary Fig. S3a). Indeed, there were no significant differences in gene expression levels of key cDC1 genes such as *IRF8*, *BATF3*, *XCR1*, and *CLEC9A* between CML patients and healthy donors (Fig. 4c). Basophils and monocytes also showed similar gene expression profiles between CML patients and healthy donors.

In contrast, remarkably different gene expression profiles were noted in CML neutrophils compared to neutrophils from healthy donors (Fig. 4a,b). Gene ontology (GO) analysis of significantly upregulated genes in CML neutrophils indicated that the genes related to the production of ROS were increased (Supplementary Fig. S3b). In addition, gene set enrichment analysis (GSEA) showed that the gene sets related to mitochondrial metabolism, such as oxidative phosphorylation and fatty acid metabolism, were highly expressed in CML neutrophils (Fig. 4d). One interpretation of these data is that ROS production is promoted through the activation of mitochondrial metabolism in CML neutrophils. It has been reported that neutrophils suppress anti-tumor immune responses via ROS generation^38–41^. Moreover, we found that CML neutrophils highly express granulocytic myeloid-derived suppressor cell (G-MDSC)-related genes such as arginase-1 (*ARG1*), myeloperoxidase (*MPO*), and *S100A8* (Fig. 4e). These results imply that CML neutrophils, which have increased populations, may contribute to compromised anti-tumor immunity.

### Monocytes in CML patients and basophils express high levels of PD-L1

**Figure 5:**
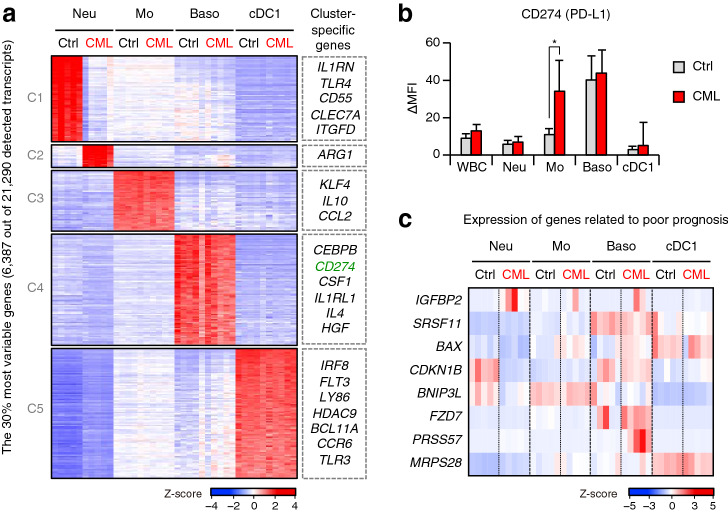
Identification of myeloid cell populations with gene expression patterns that are related to poor prognosis in CML patients. (**a**) Heat map of the top 30% differentially expressed genes among indicated myeloid cell populations from healthy controls [Ctrl] and CML patients [CML]. Gene clusters (1 to 5) were defined according to K-means clustering. Neu, neutrophils; Mo, monocytes; Baso, basophils; cDC1, type 1 conventional dendritic cells. (**b**) Protein expression levels of PD-L1 in myeloid cells of healthy controls [Ctrl] (n = 6) and CML patients [CML] (n = 14). WBC, while blood cells. Values are the mean ± SD. *P < 0.05 (Student’s *t*-test). (**c**) Heat map showing expression levels of genes related to poor prognosis in indicated myeloid cell populations from healthy controls [Ctrl] and CML patients [CML].

When we performed a cluster analysis of the top 30% differentially expressed genes (DEGs) among the control and CML myeloid cell types, we again observed that only neutrophil-related genes were divided into two distinct clusters between healthy control volunteers and CML patients (Fig. 5a and Supplementary Fig. S3c). We noticed that cluster 4, composed of basophil-specific genes, contained *CD274* (*PD-L1*) encoding the immune checkpoint molecule. Although it has been reported that PD-L1 expression is elevated in myeloid cells in CML patients and a mouse CML model^42,43^, myeloid cell types that express high levels of PD-L1 have yet to be identified. We also examined cell-surface PD-L1 protein expression in various myeloid cell types by flow cytometry. In addition to control and CML basophils, we found that CML monocytes, but not healthy control monocytes, also expressed high levels of PD-L1 (Fig. 5b), possibly via a post-transcriptional mechanism. These data suggest that high expression of PD-L1 on basophils and monocytes (also reported as monocytic-MDSCs), both of which are known to be increased in absolute numbers in CML patients^13,44^, may also contribute to compromised anti-tumor immunity.

### Basophils express genes related to poor prognosis in CML

Recently, several signature genes have been associated with poor prognosis in CML*,* including *IGFBP2*, *SRSF11*, *BAX*, *CDKN1B*, *BNIP3L*, *FZD7*, *PRSS57*, and *MRPS28*^45,46^. However, it is unclear which cell types express these genes. By examining expression of these genes in myeloid cell types in both CML patients and healthy controls, we found that most of the genes related to poor prognosis were highly expressed in basophils, especially in CML patients (Fig. 5c). These results support the long-standing clinical finding that basophil counts can indicate poor prognosis in CML.

## Discussion

In this study, we analyzed innate immune cells of the myeloid lineage, such as granulocytes, monocytes, and cDCs, as well as their progenitors in CML patients. Our data confirmed increased proportions of granulocytes (i.e., neutrophils and basophils) and reduced proportions of cDCs (i.e., cDC1s and cDC2s) and further revealed that cDC development was restricted from progenitor stages such as GMPs, MDPs, and CDPs in CML patients. Although two key DC development-related genes, *IRF8* and *FLT3*, were found to be strongly suppressed in CML cells, in vitro retroviral transduction experiments using mouse HSPCs suggested that IRF8 is particularly critical because exogenous expression of IRF8, but not FLT3, restored cDC differentiation. In addition, our data suggested that there is a hierarchy between the two factors, where IRF8 may function upstream of *FLT3*, contrary to a previous report^47^. It should be noted, however, that *IRF8*^–/–^ hematopoietic progenitor cells, such as lymphoid-primed multipotent progenitors (LMPPs) and MDPs, express normal levels of FLT3, indicating that their relationship is cell context-dependent. Given that DCs are essential to provoke CTL responses, these results illustrate how initiation of anti-tumor immune responses is compromised in CML patients.

It has been reported that decreased cDC frequency is not restored after treatment with imatinib in CML patients, suggesting that impaired anti-tumor immunity may persist long after TKI treatment^18^. We speculate that this may involve an epigenetic mechanism, which accompanies memory, for several reasons: (1) IRF8 epigenetically primes cDC-related genes in mouse LMPPs^49^, (2) IRF8 expression is reduced in HSPCs of a mouse CML model^22^ and CML patients (this study), and (3) neighboring BCR-ABL^+^ progenitors memorize the influence from BCR-ABL^+^ cells to cause a myeloid bias in CML^50^. Thus, it is tempting to envisage that even after TKI treatment, BCR-ABL^–^ cells might still be unable to efficiently express *IRF8* and generate cDCs.

We found that gene expression profiles in neutrophils are dramatically changed in patients with CML. These neutrophils appeared to augment ROS-producing potential and immunosuppressive features. Moreover, basophils and CML monocytes expressed high levels of the immune checkpoint molecule, PD-L1. Interestingly, PD-L1 expression seems to be highly expressed in both normal and CML basophils at the transcriptional level, whereas it was upregulated only in CML but not in normal monocytes at the post-transcriptional level. Notably, high PD-L1 expression in monocytes has also been reported in non-Philadelphia chromosome myeloproliferative neoplasms with a JAK2 V617F mutation via STAT3/STAT5-dependent transcriptional induction^51^. In a mouse CML model, blocking the PD-1/PD-L1 pathway is reportedly an effective treatment for CML^42,43^. Therefore, high expression of PD-L1 on basophils and monocytes may contribute to suppressing anti-tumor immune responses in CML. Furthermore, we found that basophils, whose increase is known to be one of the risk factors for poor prognosis in CML^44^, have high expression of various genes that have been previously linked with poor prognosis, again implicating basophils in CML pathogenesis.

The increase in basophils in CML patients, despite the inhibition of IRF8 by BCR-ABL, is contrary to the phenotype seen in *IRF8*^–/–^ mice, which display a severe reduction in basophil counts^52^. The discrepancy in these observations may be explained by the activation of STAT5, a target of BCR-ABL^22,53,54^. It has been shown that STAT5 induces *CEBPA*, which promotes basophil development^55^, while inhibiting *IRF8* expression^53,54,56^. Thus, we envisage that constitutively activated STAT5 in CML may override the need for IRF8 in basophil development.

In conclusion, our study provides new insights into CML pathogenesis and the basis for developing new therapies for CML involving anti-tumor immunity. Although several clinical trials on immune-activating agents, such as IFNα, an anti PD-1 antibody, and peptide vaccines have been conducted^57,58^, the effects appear to be limited. One possibility may be that it is not enough to target either an up-regulators or down-regulators of anti-tumor immunity. Further investigation into the mechanism through which BCR-ABL affects immunity would be required in order to develop a method to target both facets—restoring the “accelerator” (i.e., cDCs) and releasing the “brake” (i.e., monocytes, neutrophils, and basophils) of anti-tumor immunity.

## Materials and methods

### Human samples

Peripheral blood samples and bone marrow samples from newly diagnosed CML patients were obtained after acquiring informed consent at Yokohama City University Hospital, Yokohama City University Medical Center, Kanagawa Cancer Center, or Saiseikai Yokohama South Hospital. Peripheral blood samples from healthy volunteers were obtained after acquiring informed consent. Bone marrow samples from patients who had undergone joint surgery without hematological disorders at the Department of Orthopedics, Yokohama City University Hospital were obtained as normal controls after acquiring informed consent. Sample information is listed in Table 1 [Table 1, Patient characteristics]. We confirmed that the percentages and absolute numbers of each cell population of control individuals in our study are roughly the same as those in previous reports^48,59,60^. This study was approved by the ethics committee of Yokohama City University (A150723003). All protocols of this study conformed to the standards of Declaration of Helsinki.

### Cell isolation

For RNA sequencing (RNA-seq), cDC1s, basophils, monocytes, and neutrophils were immunostained using fluorescently labelled antibodies against cell surface markers and then isolated using a FACS Aria II (BD Biosciences) machine. The purity of the sorted populations was always above 99%. See Supplemental Materials and Methods for the list of antibodies used in this study.

### Animals

C57BL/6J mice were bred in specific pathogen-free conditions and used for experiments between 8 and 12 weeks of age. All animal experiments conformed to the ARRIVE guidelines and were conducted in accordance with the Guidelines for the Proper Conduct of Animal Experiments (Science Council of Japan), and all protocols were approved by the institutional review boards of Yokohama City University (protocols #F-A-17-018 and #F-A-20-043).

### Published data analysis

Our previously obtained microarray and RNA-seq data of DC progenitors and DC culture were retrieved from the Gene Expression Omnibus (GEO)/National Center for Biotechnology (NCBI) accession numbers GSE5550, GSE44920, GSE84509, and GSE89020^22,23,28,48^.

### Other methods

Detailed information regarding flow cytometry, retroviral transduction, conditions for culturing DCs, cell surface markers and antibodies, quantification of BCR-ABL transcript levels, RNA-seq, bioinformatics analysis, and statistical analysis are available in Supplemental Materials and Methods. RNA-seq data are accessible at the GEO/NCBI database (http://www.ncbi.nlm.nih.gov/geo/) with accession number GSE162462^22^.

## Supplementary Information

Supplementary Information.
