# Supplementary material for: Compromised anti-tumor–immune features of myeloid cell components in chronic myeloid leukemia patients
Source: Sci Rep. 2021 Sep 10;11:18046. doi: 10.1038/s41598-021-97371-8 (PMC8433374; doi:10.1038/s41598-021-97371-8)
Supplement: Supplementary file 1 — Supplementary Information. [file 41598_2021_97371_MOESM1_ESM.pdf]

SUPPLEMENTAL INFORMATION

Compromised anti-tumor–immune features of myeloid cell components in chronic myeloid leukemia patients

Ibuki Harada, Haruka Sasaki, Koichi Murakami, Akira Nishiyama, Jun Nakabayashi, Motohide Ichino, Takuya Miyazaki, Ken Kumagai, Kenji Matsumoto, Maki Hagihara, Wataru Kawase, Takayoshi Tachibana, Masatsugu Tanaka, Tomoyuki Saito, Heiwa Kanamori, Hiroyuki Fujita, Shin Fujisawa, Hideaki Nakajima and Tomohiko Tamura

SUPPLEMENTAL MATERIALS AND METHODS

Flow cytometry

Flow cytometry was performed using a FACSCanto II (BD Biosciences) cytometer, and data were analyzed using FlowJo software (FlowJo, LLC). Human peripheral blood samples or human bone marrow samples collected with heparin were hemolyzed with Pharm Lyse Lysing Buffer (BD Biosciences). Hemolyzed peripheral blood or bone marrow cells were blocked with human serum, and then immunostaining was performed against cell surface markers using fluorescently labelled antibodies. Cultured mouse dendritic cells (DCs) were subjected to IgG receptor blocking treatment, followed by cell surface immunostaining using fluorescently labelled antibodies. To detect FLT3 on FLT3L-treated cells, the cells were washed twice followed by incubation at 37°C for 1 hour with an FLT3L-free medium.

Retroviral transduction and *in vitro* culture of DCs

Retroviral transduction and *in vitro* culture of DCs were performed as previously described.[1] The following murine stem cell virus (MSCV) retroviral vectors were used: MIG [MSCV-internal ribosome entry site (IRES)-GFP], MIG-p210$^{BCR\text{-}ABL}$ (MSCV-p210$^{BCR\text{-}ABL}$-IRES-GFP), MICD8 [MSCV-IRES-human truncated CD8 (hCD8t)], MICD8-IRF8 (MSCV-IRF8-IRES-hCD8t), and MICD8-FLT3 (MSCV-FLT3-IRES-hCD8t). After the mice were euthanized, the femurs and tibias were removed and flushed to obtain bone marrow cells.

Bone marrow lineage marker-negative (Lin$^-$) cells were purified by the MACS system using the Lineage Cell Depletion Kit (Miltenyi Biotec), and transduced with MSCVs by spinoculation for 2 consecutive days. MSCVs were washed 24 h after the last spinoculation and cultured with 100 ng/mL human FLT3-ligand (FLT3L) for 7 days. All cytokines were purchased from PeproTech.

Cell surface markers and antibodies

Human samples: For staining lineage marker-positive (Lin$^+$) cells, the Lineage Cell Detection Cocktail (Miltenyi Biotec) was used, containing antibodies against CD5, B220/CD45R, CD11b, Gr-1, 7–4, and Ter-119. In addition, 7-amino-actinomycin D was used to detect and exclude dead cells. Cell surface markers used for flow cytometric analysis and cell sorting in this study are as follows[2-4]: CMPs, Lin$^-$ CD34$^+$ CD38$^+$ CD135$^+$ CD45RA^{low}; MEPs, Lin$^-$ CD34$^+$ CD38$^+$ CD135$^-$ CD45RA^{low}; GMPs, Lin$^-$ CD34$^+$ CD38$^+$ CD135$^+$ CD45RA^{high}; GMDPs, Lin$^-$ CD34$^+$ CD38$^+$ CD123^{low} CD45RA$^+$ CD115$^-$; MDPs, Lin$^-$ CD34$^+$ CD38$^+$ CD45RA$^+$ CD115$^+$; CDPs, Lin$^-$ CD34$^+$ CD38$^+$ CD123^{high} CD45RA$^+$ CD115$^-$; Neutrophils (Neu), CD66b$^+$; Monocytes (Mo), CD14$^+$; Basophils (Baso), CD123$^+$ CD203c$^+$; B cells, CD19$^+$; T cells, CD3e$^+$; cDC1, CD3e$^-$ CD19$^-$ CD303$^-$ CD141$^+$ CD1c$^-$; cDC2, CD3e$^-$ CD19$^-$ CD303$^-$ CD141$^-$ CD1c$^+$. Intracellular IRF8 staining was performed as previously described.[5] Briefly, CD34$^+$ cells were fixed with 4% paraformaldehyde, treated with permeabilizing solution (50 mM NaCl, 5 mM EDTA, and 0.5% Triton X-100), and stained with a monoclonal anti-IRF8 (U31-644) antibody from BD Biosciences. Clone names and antibodies used are as

follows: anti-CD38 (HB-7), anti-CD135 (BV10A4H2), anti-CD123 (6H6), anti-CD14 (63D3), anti-CD66b (G10F5), anti-CD141 (M80), anti-CD1c (L161), anti-CD3e (APA1/1), anti-CD19 (4G7), anti-CD115 (9-4D2-1E4), anti-CD303 (201A), anti-CD45RA (HI100), anti-CD203c (NP4D6), which were obtained from BioLegend. Anti-CD34 (4H11) antibody was obtained from eBioscience.

Mouse dendritic cells: The cell surface marker for cDCs used in flow cytometric analysis was CD11c^{+} MHC II^{+1}: Intracellular IRF8 was stained as described above. Antibodies for murine DCs and their clone names were anti-CD11c (N418), anti-MHC-class II (AF6-120.1), and anti-CD135 (A2F10), which were obtained from BioLegend. Anti-IRF8 (U31-644) was obtained from BD Biosciences.

Quantification of *BCR-ABL* transcript levels

To determine BCR-ABL transcript levels in human cDC1s, basophils, monocytes, and neutrophils, TaqMan Assays (Thermo Fisher, assay ID Hs03024541_ft) were performed according to the manufacturer's instructions. Data were analyzed based on a previously described method.[6] Briefly, the limit of detection (LOD) was determined from the Ct value, and the expression levels of BCR-ABL relative to the LOD in each cell type were calculated.

RNA-sequencing

We adapted the method previously reported by Giustacchini et al.[6] cDNA was used for the tagmentation reaction carried out with the Nextera XT DNA Sample Preparation kit

(Illumina) according to the manufacturer's instructions. The product was purified with a 1:1 ratio of AMPure XP beads, with a final elution in resuspension buffer provided from the Nextera kit. Samples were loaded on a High-Sensitivity DNA chip (Agilent Technologies) to check the size and quality of the indexed library, and the concentration was measured with a Qubit High-Sensitivity DNA kit (Invitrogen). All samples were confirmed to be suitable for sequencing based on the size of the indexed cDNA library. Libraries were sequenced with an Illumina HiSeq 500 (75 bp single-end read) at the Advanced Medical Research Center in Yokohama City University. RNA-seq analyses were performed with a sequencing depth of 20 million reads per sample.

Bioinformatics analysis

FASTQ-formatted RNA-seq data were mapped onto the human reference genome GRCh38 using TopHat software.[7] FPKMs were calculated using Cufflinks.[7] PCA was performed using the prcomp function in R. Pearson's correlation coefficients were calculated to obtain correlation matrices. For gene ontology (GO) analysis, differentially expressed genes (DEGs) were identified using the edgeR package (R version 3.1.2) with FDR < 0.01 and logFC > 2 in each cell type between healthy controls and CML patients. The list of DEGs was applied for the BiNGO App of Cytoscape[8] as a query. GO terms enriched in this DEG list were ranked by p-value, and the top five enriched terms were listed. Gene set enrichment analysis (GSEA) was performed using the GSEA (version 2.0) software available at (http://www.broad.mit.edu/gsea).[9] Expression levels of each gene were quantified by Z-scores, and DEGs were identified using the limma package (R

version 3.1.2) with FDR < 0.05. The top 30% of DEGs were clustered according to K-means clustering. The obtained result was displayed as a heatmap using Java TreeView.[10] Then, the represented genes, including CD247, were manually mapped using a heatmap. The expression levels of genes associated with disease progression[6, 11-16] were converted to Z-scores and shown with a heatmap. Z-scores were calculated as previously described.[17]

Statistical analysis

Statistical significance of data was analyzed by the unpaired t test or ANOVA, after testing for normal distribution, unless indicated otherwise. *P* value is shown as: *, $P < 0.05$; **, $P < 0.01$; and ***, $P < 0.001$.

Data Availability

The data supporting the findings of this study are available from the corresponding author upon reasonable request. The sequencing data generated in this study were deposited in the Gene Expression Omnibus database (accession number: GSE162462; the secure token for reviewers is “crivuccclnondwj”).

References for Supplemental Materials and Methods

1 Watanabe T, Hotta C, Koizumi S, et al. The transcription factor IRF8 counteracts BCR-ABL to rescue dendritic cell development in chronic myelogenous leukemia. *Cancer research*. 2013; 73: 6642-6653.

2 Breton G, Lee J, Zhou YJ, et al. Circulating precursors of human $CD1c^{+}$ and $CD141^{+}$ dendritic cells. *The Journal of experimental medicine*. 2015; 212: 401-413.

3 Breton G, Lee J, Liu K, Nussenzweig MC. Defining human dendritic cell progenitors by multiparametric flow cytometry. *Nature protocols*. 2015; 10: 1407-1422.

4 Bruck O, Blom S, Dufva O, et al. Immune cell contexture in the bone marrow tumor microenvironment impacts therapy response in CML. *Leukemia*. 2018; 32: 1643-1656.

5 Sasaki H, Kurotaki D, Osato N, et al. Transcription factor IRF8 plays a critical role in the development of murine basophils and mast cells. *Blood*. 2015; 125: 358-369.

6 Giustacchini A, Thongjuea S, Barkas N, et al. Single-cell transcriptomics uncovers distinct molecular signatures of stem cells in chronic myeloid leukemia. *Nature medicine*. 2017; 23: 692-702.

7 Trapnell C, Roberts A, Goff L, et al. Differential gene and transcript expression analysis of RNA-seq experiments with TopHat and Cufflinks. *Nature protocols*. 2012; 7: 562-578.

8 Maere S, Heymans K, Kuiper M. BiNGO: a Cytoscape plugin to assess overrepresentation of gene ontology categories in biological networks. *Bioinformatics (Oxford, England)*. 2005; 21: 3448-3449.

9 Subramanian A, Tamayo P, Mootha VK, et al. Gene set enrichment analysis: a knowledge-based approach for interpreting genome-wide expression profiles. *Proceedings of the National Academy of Sciences of the United States of America*. 2005; 102: 15545-15550.

10 Saldanha AJ. Java Treeview--extensible visualization of microarray data. *Bioinformatics (Oxford, England)*. 2004; 20: 3246-3248.

11 Kok CH, Yeung DT. Gene expression signature that predicts early molecular response failure in chronic-phase CML patients on frontline imatinib. 2019; 3: 1610-1621.
12 Agarwal A, Mackenzie RJ, Besson A, et al. BCR-ABL1 promotes leukemia by converting p27 into a cytoplasmic oncoprotein. *Blood*. 2014; 124: 3260-3273.
13 Radich JP, Dai H, Mao M, et al. Gene expression changes associated with progression and response in chronic myeloid leukemia. *Proceedings of the National Academy of Sciences of the United States of America*. 2006; 103: 2794-2799.
14 McWeeney SK, Pemberton LC, Loriaux MM, et al. A gene expression signature of $CD34^+$ cells to predict major cytogenetic response in chronic-phase chronic myeloid leukemia patients treated with imatinib. *Blood*. 2010; 115: 315-325.
15 Villuendas R, Steegmann JL, Pollán M, et al. Identification of genes involved in imatinib resistance in CML: a gene-expression profiling approach. *Leukemia*. 2006; 20: 1047-1054.
16 Diaz-Blanco E, Bruns I, Neumann F, et al. Molecular signature of CD34(+) hematopoietic stem and progenitor cells of patients with CML in chronic phase. *Leukemia*. 2007; 21: 494-504.
17 Kurotaki D, Nakabayashi J, Nishiyama A, et al. Transcription Factor IRF8 Governs Enhancer Landscape Dynamics in Mononuclear Phagocyte Progenitors. *Cell reports*. 2018; 22: 2628-2641.

Supplemental Figure 1. Gating strategy.

(a) Representative FACS plots of immune cells in peripheral blood. (b) Representative FACS plots of myeloid progenitors in bone marrow.

Supplemental Figure 2. Expression of key molecules in human and mouse CML cells.

(a) Expression of *DNTT*, *IRF8*, and *FLT3* in human DC progenitors. (b) Expression of *IRF8* and *FLT3* in $CD34^+$ cells of CML patients, and of *Irf8* and *Flt3* in mouse bone marrow Lin^- cells transduced with BCR-ABL *in vitro* for 3 days. (c) Histograms of FLT3 protein expression on unwashed or washed (twice, followed by incubation at 37°C for 1 hour with an FLT3L-free medium) cells that had been generated by *in vitro* bone marrow cell culture in the presence of FLT3L for 4 days. Data are representative of 3 similar experiments. (d) Expression of IRF8 and FLT3 proteins in *in vitro* mouse DC culture co-transduced with BCR-ABL and IRF8 or FLT3 (n = 3). Data were retrieved from GSE89020, GSE5550, and GSE44920. All values are expressed as mean ± SD. **P* < 0.05, ***P* < 0.01, ****P* < 0.001 (Student's *t*-test).

Supplemental Figure 3. Transcriptomic analysis of myeloid cells in CML patients.

(a) TaqMan qPCR assay for *BCR-ABL* mRNA expression. LOD, limit of detection. (b) Gene ontology (GO) analysis of genes upregulated in CML neutrophils. (c) GO biological-process terms enriched in each cluster identified in Figure 4a. (d) *CD274 (PD-*

L1) mRNA expression in myeloid cells from healthy controls [Ctrl] and CML patients [CML]. Values in (a) and (d) are the mean ± SD. *$P < 0.05$, **$P < 0.01$ (Student's *t*-test).

Supplemental Figure 1

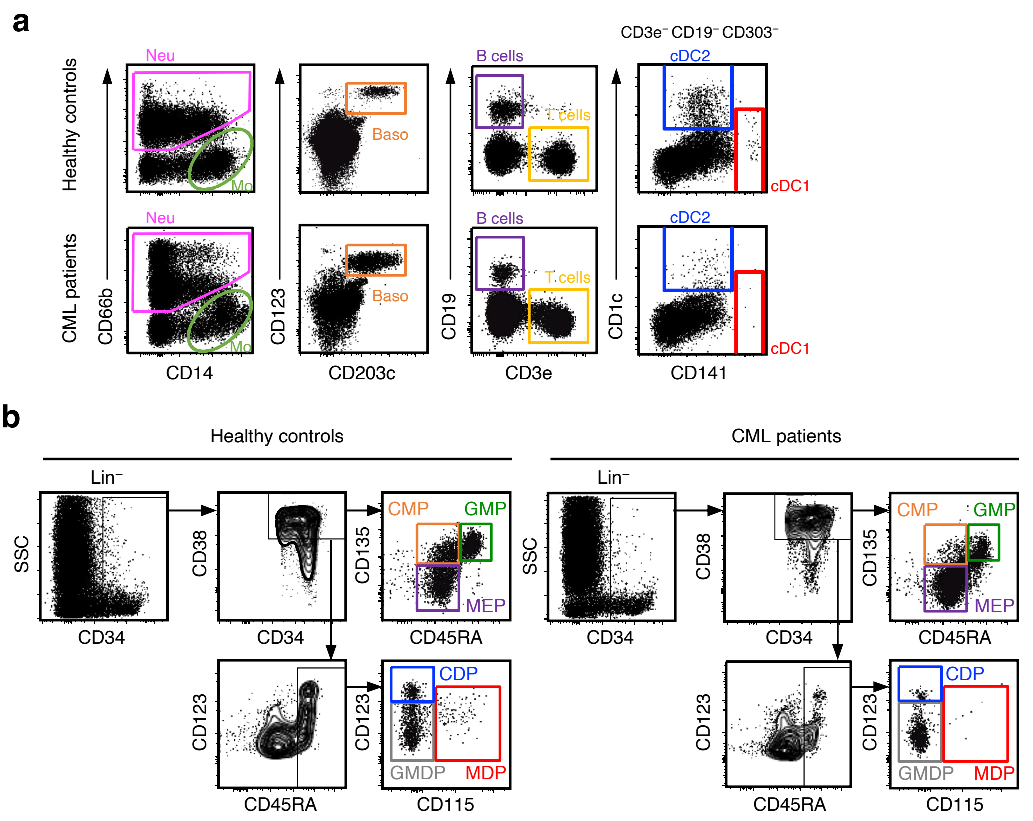

Supplemental Figure 1. Gating strategy.
(a) Representative FACS plots of immune cells in peripheral blood. (b) Representative FACS plots of myeloid progenitors in bone marrow.

Supplemental Figure 2

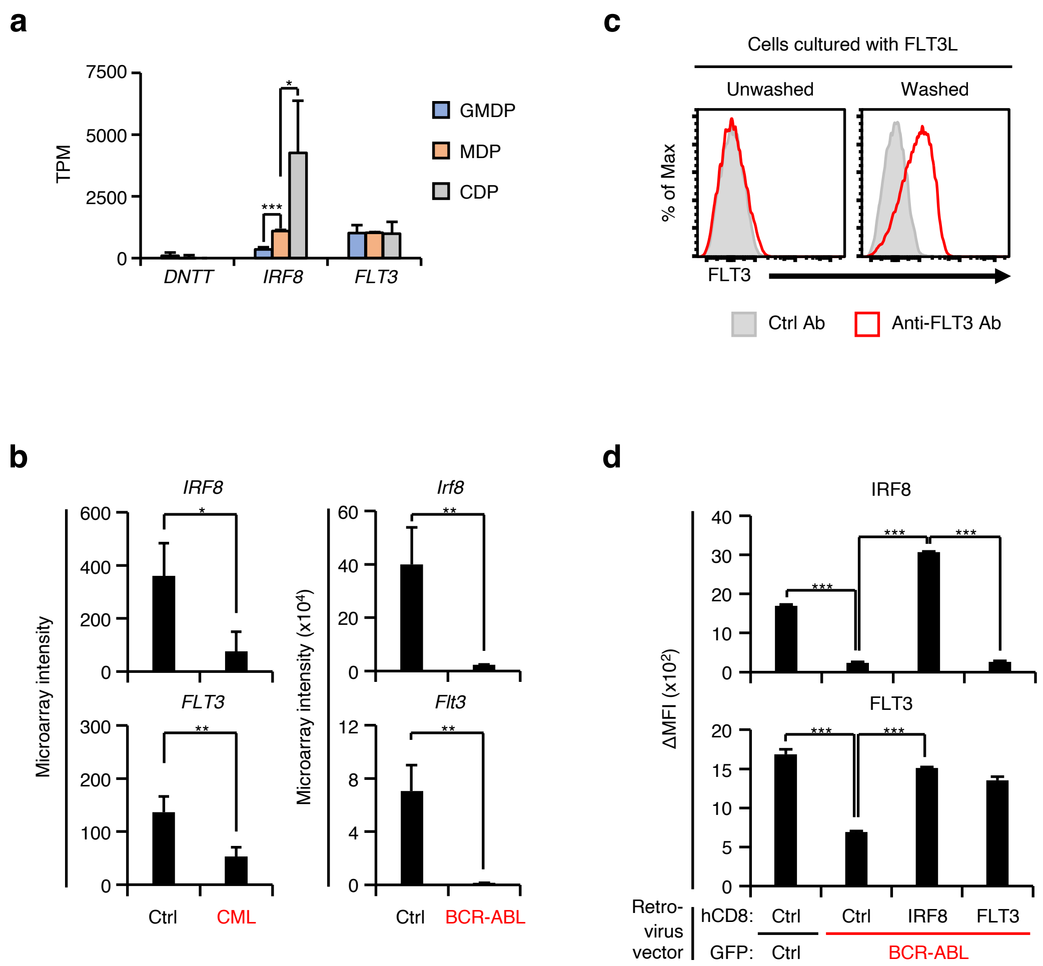

Supplemental Figure 2. Expression of key molecules in human and mouse CML cells. (a) Expression of *DNTT*, *IRF8*, and *FLT3* in human DC progenitors. (b) Expression of *IRF8* and *FLT3* in $CD34^+$ cells of CML patients, and of *Irf8* and *Flt3* in mouse bone marrow Lin^- cells transduced with BCR-ABL *in vitro* for 3 days. (c) Histograms of FLT3 protein expression on unwashed or washed (twice, followed by incubation at 37°C for 1 hour with an FLT3L-free medium) cells that had been generated by *in vitro* bone marrow cell culture in the presence of FLT3L for 4 days. Data are representative of 3 similar experiments. (d) Expression of IRF8 and FLT3 proteins in *in vitro* mouse DC culture co-transduced with BCR-ABL and IRF8 or FLT3 (n = 3). Data were retrieved from GSE89020, GSE5550, and GSE44920. All values are expressed as mean ± SD. **P* < 0.05, ***P* < 0.01, ****P* < 0.001 (Student's *t*-test).

Supplemental Figure 3

a

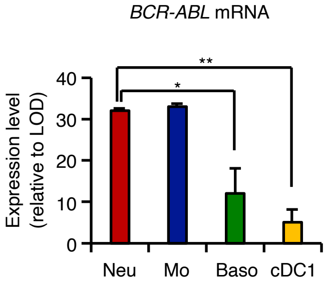

b

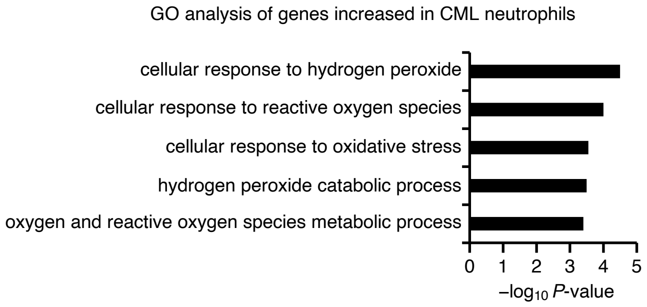

c

C1

GO term	*P*-value
immune system process	2.4×10^{-16}
signal transduction	6.3×10^{-15}
signaling	2.7×10^{-14}
immune response	4.0×10^{-14}
signaling pathway	1.2×10^{-13}

C2

GO term	*P*-value
defense response	3.8×10^{-14}
killing of cells of another organism	2.0×10^{-11}
defense response to fungus	9.2×10^{-11}
cell killing	1.5×10^{-10}
response to fungus	4.9×10^{-10}

C3

GO term	*P*-value
cellular membrane organization	1.3×10^{-11}
membrane organization	1.5×10^{-11}
membrane invagination	1.6×10^{-11}
endocytosis	1.6×10^{-11}
vesicle-mediated transport	9.6×10^{-11}

C4

GO term	*P*-value
regulation of metabolic process	9.7×10^{-12}
cellular macromolecule metabolic process	1.1×10^{-11}
protein modification process	1.2×10^{-11}
regulation of cellular metabolic process	1.3×10^{-11}
regulation of cellular process	1.3×10^{-11}

C5

GO term	*P*-value
translation	2.6×10^{-74}
cellular metabolic process	1.6×10^{-68}
gene expression	1.9×10^{-67}
translational elongation	3.4×10^{-64}
metabolic process	1.8×10^{-54}

d

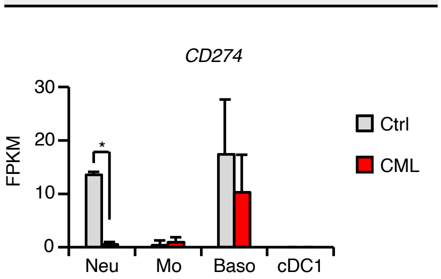

Supplemental Figure 3. Transcriptomic analysis of myeloid cells in CML patients.
(a) TaqMan qPCR assay for *BCR-ABL* mRNA expression. LOD, limit of detection. (b) Gene ontology (GO) analysis of genes upregulated in CML neutrophils. (c) GO biological-process terms enriched in each cluster identified in Figure 4a. (d) *CD274 (PD-L1)* mRNA expression in myeloid cells from healthy controls [Ctrl] and CML patients [CML]. Values in (a) and (d) are the mean ± SD. *$P < 0.05$, **$P < 0.01$ (Student's *t*-test).
